# Therapeutic Strategies for Infectious Multiple Aortic Aneurysms: Thoracoabdominal Aortic Aneurysm Treatment Using a Fenestrated Stent-Graft

**DOI:** 10.3400/avd.cr.20-00032

**Published:** 2020-09-25

**Authors:** Satoshi Okugi, Takashi Azuma, Yoshihiko Yokoi, Satoru Domoto, Hiroshi Niinami

**Affiliations:** 1 Department of Cardiovascular Surgery, The Tokyo Women’s Medical University

**Keywords:** aorta, thoracoabdominal aortic aneurysm, endovascular surgery, stent graft, case report

## Abstract

Recently, it has been reported that a fenestrated stent graft is an effective option in the treatment of pararenal artery abdominal aortic aneurysm. We report the case of a 72-year-old male patient with multiple aortic aneurysms in the distal arch, thoracoabdominal aorta, right common iliac artery, as well as a pararenal abdominal aortic aneurysm. The patient was found to have a mass with a tendency of rapid expansion within a month from its discovery. Because it was a saccular aneurysm with a tendency of rapid expansion and wide spread, the risk of rupture was judged to be high, and surgical treatment became necessary. One-stage treatment was desirable; therefore, endovascular treatment with a fenestrated stent graft was selected. Four fenestrations were made to a stent graft for the celiac artery, superior mesenteric artery, and bilateral renal arteries. The postoperative computed tomography (CT) showed no branch occlusion or endoleak, and the 2-year postoperative CT showed the shrinkage and subsequent disappearance of the aortic aneurysm at the treatment site. For extensive aortic aneurysm, including pararenal artery abdominal aortic aneurysms, one-stage treatment with fenestrated stent graft was considered to be effective as a treatment strategy. (This is a translation of Jpn J Vasc Surg 2020; 29: 9–13.)

## Introduction

It remains debatable whether open surgery or endovascular aneurysm repair should be used for pararenal abdominal aortic aneurysms. Nevertheless, treatment with a fenestrated stent graft has been reported as an effective option. Extensive aortic aneurysms cannot be treated simply because they often require multi-stage surgeries to minimize risks and invasiveness. Furthermore, because of underlying diseases and complications, making decisions regarding treatment strategies becomes more difficult. We encountered a case of rapidly growing, extensive, infectious multiple aortic aneurysms, including a pararenal abdominal aortic aneurysm that was treated with a custom fenestrated stent graft during one-stage treatment.

## Case Report

A 72 year-old man with generalized fatigue was hospitalized at our Department of Diabetes Medicine because of rapidly worsening blood sugar control and an increased inflammatory response. He had a history of hypertension and type II diabetes mellitus.

Characteristics on admission were as follows: height, 173 cm, and body weight, 59 kg. Hematological findings at the time of the initial examination were as follows: white blood cell count, 10190/µL; neutrophils, 82.5%; C-reactive protein (CRP), 7.24 mg/dL; creatinine (Cr), 2.25 mg/dL; and hemoglobin A1c, 9.3%. These results indicated a high leukocyte count and inflammatory response, low renal function, and poor diabetes control.

**Figure 1:**
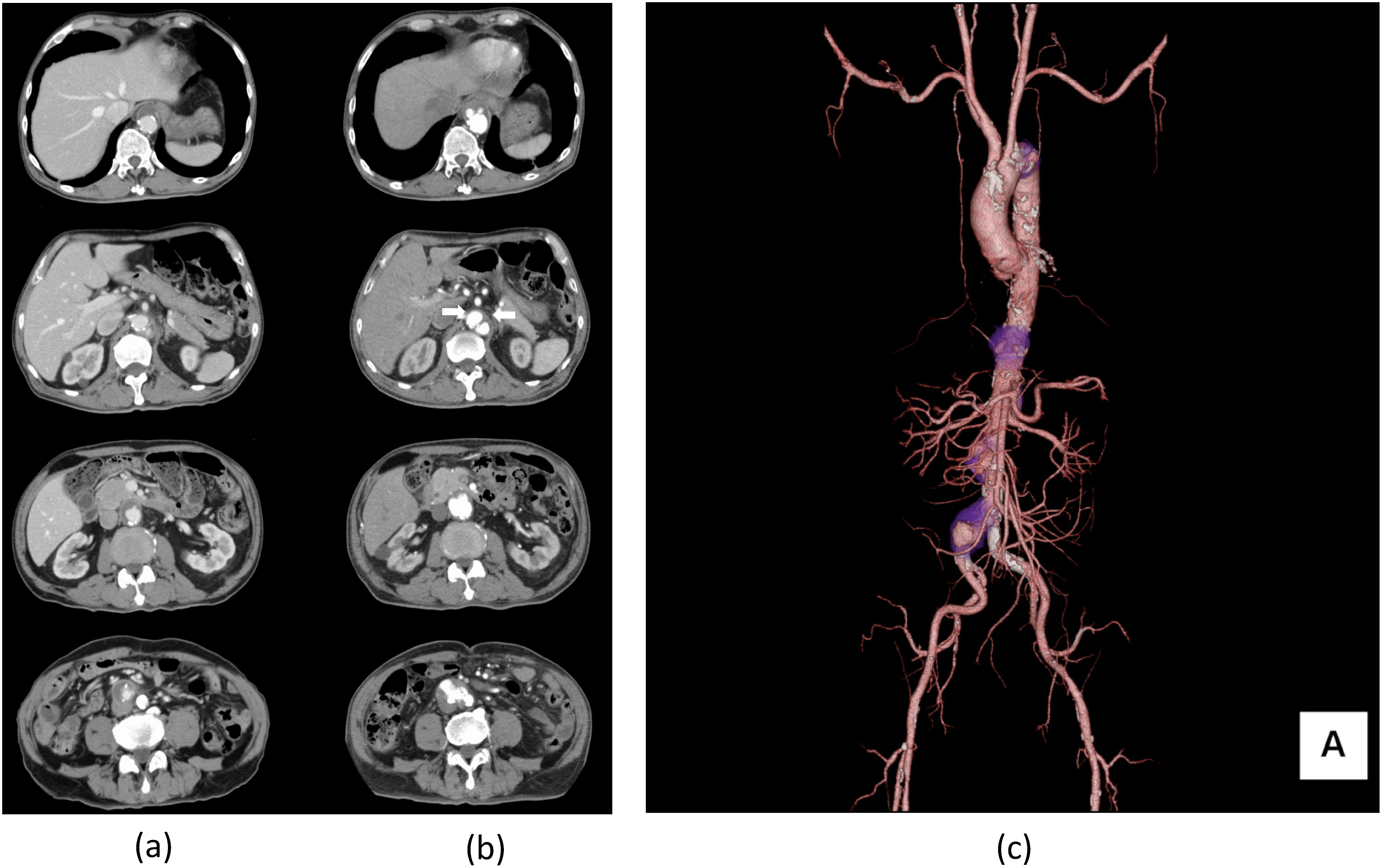
Fig. 1 Contrast-enhanced computed tomography (CT) examination at admission (**a**) and one month later (**b**) and (**c**) show multiple aortic aneurysms and rapid enlargement of the aneurysm diameter. The portion corresponding to the sealing zone between the fenestration of the celiac artery and the superior mesenteric artery and the saccular aneurysm is indicated by arrows.

Contrast-enhanced computed tomography (CT) showed localized dissection from the abdominal aorta to the right common iliac artery. He was suspected of having an infectious abdominal aortic aneurysm and was referred to our department (**Fig. 1a**).

After admission, antibiotic therapy (vancomycin, ceftriaxone, and levofloxacin) was started for the suspected infectious aortic aneurysm. Consequently, the leukocyte count and CRP decreased steadily (CRP, 7.24 to 0.12). Blood culture tests were conducted multiple times, and all yielded negative results. However, the antibiotic therapy was continued for a total of 6 weeks, following which the inflammation resolved.

Contrast CT conducted 1 month later showed a penetrating aortic ulcer-like irregular inflow of contrast agent in the lumen of the aortic aneurysm. Furthermore, the aortic aneurysm had enlarged compared with the CT imaging on admission (descending aorta, 36–43 mm; suprarenal aorta, 37–44 mm; infrarenal aorta, 34–37 mm; and right common iliac artery, 31–35 mm), thus indicating the necessity for the treatment (**Figs. 1b** and **1c**).

Because of his advanced age, poorly controlled diabetes mellitus, and renal dysfunction, staged surgery was considered adequate for treating his extensive aortic aneurysms. However, saccular aneurysm was observed to be growing rapidly. As the infection was controlled, a one-step treatment with a fenestrated stent graft was considered better.

Endovascular surgery was conducted in the supine position under general anesthesia. The common femoral arteries (FA) were exposed bilaterally. A 12-Fr safe sheath was placed in the right FA, and a 6-Fr sheath was placed in the left FA. A hybrid operating room (Trinias; Shimadzu Corporation, Kyoto, Japan) was used for angiography.

The Zenith TX2 Extension (ESBE-26-80-T-PF; Cook Medical Inc., Bloomington, IN, USA) was removed from the sheath. Based on the CT image, four fenestrations of 10, 13, 10, and 10 mm were created for the 7 mm celiac artery, 7 mm superior mesenteric artery (SMA), 5 mm left renal artery (RA), and 5 mm right RA, respectively.

**Figure 2:**
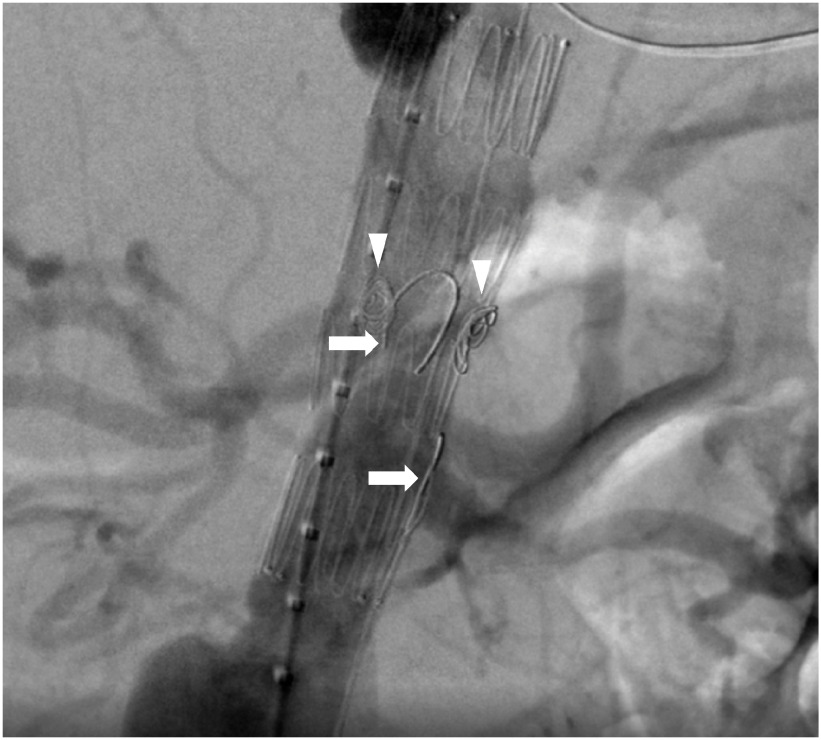
Fig. 2 Intraoperative fluoroscopic image shows snares for reinforcement of the superior mesenteric artery and left renal artery fenestration (arrows) and tornado coils to prevent endoleak from the fenestrations (triangles).

To prevent endoleaks from the fenestrations, two 4/8 mm Tornado coils (MWCE-18S-4/8-TORNADO-LEF-081800; Cook Medical Inc.) were deployed at the portion sealing zone between the fenestrations for the celiac artery, SMA, and the saccular aneurysms (**Fig. 1**). Then, they were gathered into a 10 mm bundle and sutured with a single interrupted 6-0 polypropylene suture (**Fig. 2**) to prompt thrombosis at the same site, thereby reinforcing attachment between the wall of the aorta and surrounding areas. To ensure that the fenestration site under fluoroscopy can be visualized more easily and that the fenestration opened in circular shape without deformation, the metal portions of Amplatz Goose neck snares (Medtronic, Dublin, IE) were sutured to the fenestrations for the SMA and left RA with 6-0 polypropylene sutures (**Fig. 2**) and then re-inserted in the sheath.

A pigtail catheter and Radiofocus Guidewire (Terumo, Tokyo, Japan) were inserted through the right FA to replace the guidewire with a Lunderquist Extra-Stiff Wire Guide (Cook Medical Inc.) at the ascending aorta. The TX2 Extension (ESBE-26-80-TPF) fenestrated beforehand was inserted from the right FA and deployed after ensuring its position with the contrast image (**Fig. 2**). To completely exclude the thoracoabdominal aortic aneurysm, another TX2 Extension (ESBE-28-80-T-PF) was deployed. Endologix Powerlink® (EPL) stent (28-16-120BL; Endologix, Irvine, CA, USA) was inserted via the right FA and deployed to sit on the terminal aorta. An additional EPL Extension (28-28-75L0) was deployed and adjusted with a coda balloon (32 mm; Cook Medical Inc.) to bridge the gap between the EPL main body and the fenestrated TX2 Extension.

The final imaging procedure was conducted to ensure that there were no occluded branches or endoleaks so that the surgery could be completed. The operative time was 215 min, with a blood loss of 67 mL; 200 mL of contrast agent was used postoperatively, and the patient was extubated in the operating room and transferred to the intensive care unit. Oral tranexamic acid was administered postoperatively. Cefazolin was administered for 48 h as perioperative antibiotic therapy, and then, it was discontinued.

**Figure 3:**
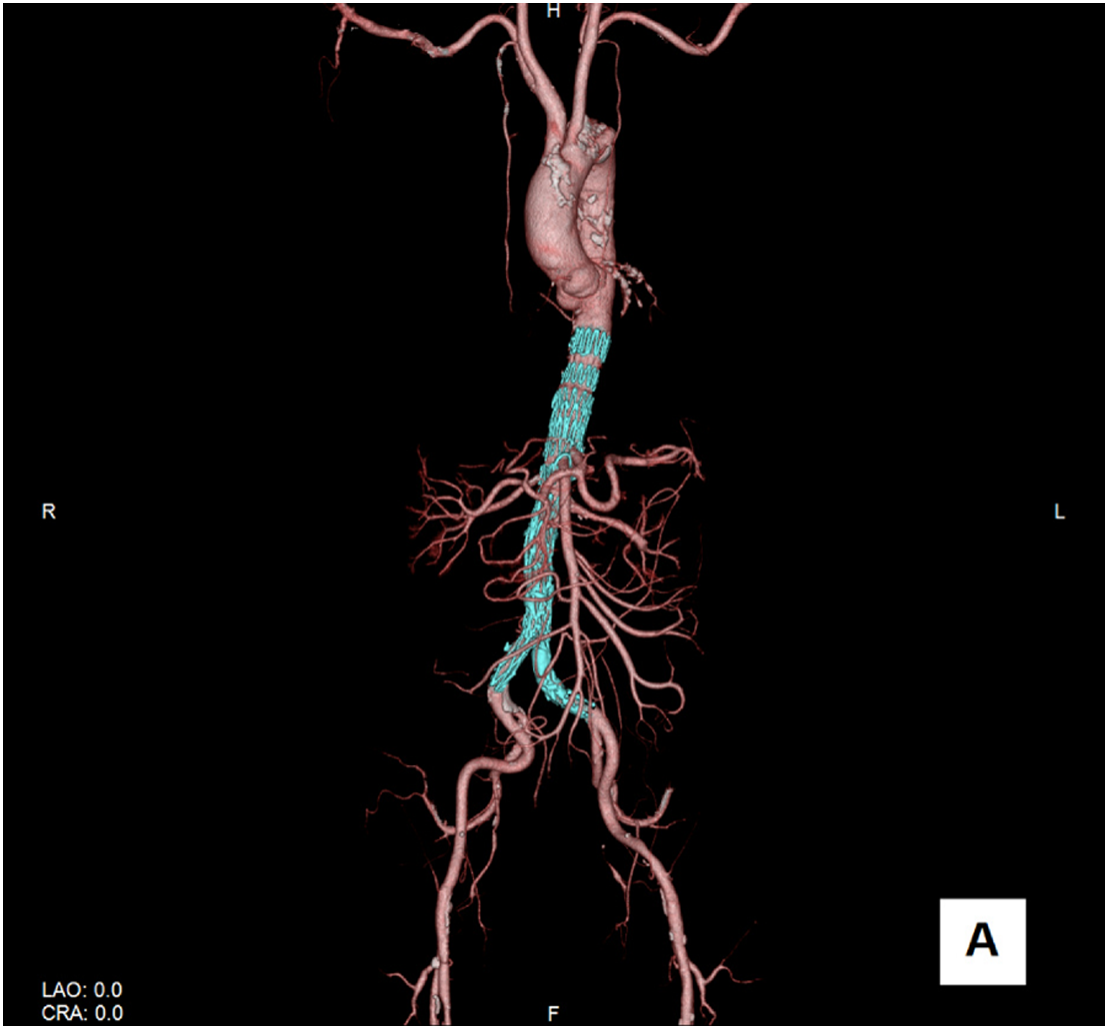
Fig. 3 Postoperative contrast-enhanced computed tomography (CT) examination shows no endoleak and patency of the abdominal branches.

The postoperative course was favorable. According to CT conducted on day 7 postoperatively, no occlusions or endoleaks at the branches occurred. The patient was able to walk independently at the time of discharge on day 8 postoperatively (**Fig. 3**). The recent follow-up CT at 2 years and 4 months postoperatively indicated shrinkage and disappearance of the aortic aneurysms at the treated site. Furthermore, there were no recurrent findings of infection during the follow-up period.

## Discussion

There are many recent reports of thoracoabdominal aortic aneurysms treated by fenestrated stent grafts; however, experts are not in agreement about whether to perform open replacement of an aneurysm with a synthetic graft or to select a stent graft for a thoracoabdominal aortic aneurysm. The patient described here had multiple aortic aneurysms, including a pararenal abdominal aortic aneurysm; therefore, stent graft treatment required fenestrations. We observed a successful treatment in a one-stage surgery with fenestrated stent grafts. Moreover, the follow-up showed the complete disappearance of aneurysms with no recurrence of infections. The 30 day mortality rate for open surgery for pararenal abdominal aortic aneurysms is higher than that for stent graft therapy.^1)^ Ou et al. reported the safety and effectiveness of fenestrated stent grafts for pararenal abdominal aortic aneurysms in high-risk cases.^2)^ Shahverdyan et al. reported that the fenestrated stent graft treatment for pararenal abdominal aortic aneurysms did not result in higher postoperative mortality or renal dysfunction rates than open surgery.^3)^ Other studies have also reported the efficacy of fenestrated and branched stent grafts for pararenal abdominal aortic aneurysms.^4,5)^

The patient described here had extensive multiple aortic aneurysms from the descending thoracic aorta to the quadrifurcation of the abdominal aorta and right common iliac artery; consequently, synthetic graft replacement of the aneurysms for a large segment of the aorta was required, from the thoracoabdominal aorta to the common iliac artery. Staged surgery was avoided because the aneurysm diameter was growing rapidly, and the aortic aneurysm was saccular. Despite being highly invasive and high-risk surgery, we determined one-stage therapy suitable in the present case. Stent graft treatment was possible because, anatomically, the patient was a good candidate with no severe curves of the aorta and there was no stenosis or severe calcification that would prevent access. Although blood culture results were negative, the patient was suspected of having an infectious aortic aneurysm based on the course and aortic aneurysm morphology. However, the normalized leukocyte count and CRP became negative with antibiotic therapy indicating that the infection was cleared. Infectious aortic aneurysms are associated with high mortality, with reported rates of 23.5% to 37%.^6)^ Diabetes mellitus is a risk factor for infectious aortic aneurysms that are caused mainly by Gram-positive coccus and Gram-negative bacillus species. They often form saccular aneurysms that expand rapidly. Kan et al. reported that the infection was controlled in 77% of the 48 patients treated with stent grafts for thoracic or abdominal aortic aneurysms and that the 30 day mortality and 2 year overall survival rates were 10.4% and 82.2%, respectively. Good outcomes (2 year overall survival, 94%) were observed in patients with controlled infection.^7)^

As mentioned, some reports have supported stent graft therapy for infectious abdominal aortic aneurysms. In this case, there was a risk of an infectious aortic aneurysm. However, we waited to confirm the negative inflammatory response after antibiotic treatment before proceeding with surgery, which likely contributed to the good outcome. Additionally, multiple saccular aneurysms are morphological characteristics of infectious aortic aneurysms. Since saccular aneurysms are less prone to stent graft migration and consequent branch occlusion, the use of fenestrated stent grafts is feasible. Moreover, the vessels are too small for branched stent grafts and for the reconstruction of the branches. Therefore, fenestrated stent grafts are advantageous because they do not require branch reconstruction. One of the few risks is occlusion of the branch by the fenestrated portion during placement, but it was placed almost exactly as planned in the present patient and could be conducted similarly in others with negligible age-related angiopathy or aortic curvature. Furthermore, the treatment for this patient was possible because the fenestrations were conducted by a highly skilled operator using elaborate imaging analysis and because the stent graft was placed in an accurate location; this is not something that can be easily imitated. The procedure requires a highly skilled operator with extensive experience. Moreover, it is best conducted using a hybrid operating room equipped with a fluoroscope. No recurrence of infection occurred during follow-up; nevertheless, continued careful follow-up and observations are necessary.

## Conclusion

We encountered a patient with extensive multiple infectious aortic aneurysms in the descending thoracic aorta and quadrifurcation of the abdominal aorta and right common iliac artery. We treated the patient with one-stage surgery with a custom stent graft created by fenestrating a commercially made stent graft. One-stage therapy with a fenestrated stent graft is an effective strategy for extensive aortic aneurysms that cannot be treated with open surgery due to the high risk.

## Additional Remarks

This study was presented at the 46th Annual Meeting of Japanese Society for Vascular Surgery (May 10, 2018, Yamagata, Japan).

This study was approved by the ethics committee of the present institution (“*Tashisetsu kyoudou ni yoru kyoubu daidoumyakuryu ni okeru shounin kiki wo mochiita Physician-Modified Endograft sutento gurafuto chiryou seiseki no kentou* [Multicenter joint investigation of therapeutic outcomes of physician-modified endograft stent-graft using approved devices for thoracic aortic aneurysm]”; approval no. 5287).

## Notes

This is a translation of Jpn J Vasc Surg 2020; 29: 9–13.
